# Preparation of Crosslinked Alginate Hydrogels for the Adsorption and Sustainable Release of Doxorubicin Hydrochloride

**DOI:** 10.3390/polym17243294

**Published:** 2025-12-12

**Authors:** Huda O. Bahwal, Kalsoom Akhtar, Wafa A. Bawazir, Shouq H. Alharthi, Sher Bahadar Khan

**Affiliations:** Chemistry Department, Faculty of Science, King Abdulaziz University, P.O. Box 80203, Jeddah 21589, Saudi Arabia; hbahwal0003@stu.kau.edu.sa (H.O.B.); wbawazeer@kau.edu.sa (W.A.B.); salharthi0393@stu.kau.edu.sa (S.H.A.); sbhkhan@kau.edu.sa (S.B.K.)

**Keywords:** hydrogel, alginate, adsorption, doxorubicin hydrochloride, drug delivery

## Abstract

Since it is expensive and takes considerable time to synthesize a new drug or improve an old one, a drug carrier can be used instead for control and targeted release of the drug. In this study, hydrogel beads were used as drug carriers for the controlled release of doxorubicin hydrochloride. Aiming to incorporate doxorubicin hydrochloride into hydrogel, various metal crosslinked alginate beads were prepared. Doxorubicin hydrochloride was incorporated by adsorption into the beads and studied the factors affecting the adsorption of drug onto the hydrogel beads. The results showed that ferric crosslinked alginate (Fe(III)–Alg) and stannous crosslinked alginate (Sn-Alg) hydrogel beads had a better adsorption percentage which was more than 21%. The amount of hydrogel, time, drug concentration, and pH of the solution all influenced the adsorption percentage. Hence, the adsorption was the best at neutral pH after 24 h when 100 mg of Fe(III)–Alg was added to the drug. Moreover, the release of the drug at different body simulation pH was investigated. The time and pH of the solution influenced the drug release where maximum drug release percentage was 82.822% after 25 h when the solution’s pH was 1.52. This system is assumed to follow the Higuchi kinetic release model.

## 1. Introduction

Since improving the ratio of safety-to-efficacy of old drugs and creating new therapeutics are costly and time-consuming, controlled drug delivery systems (DDSs) are an appealing method and have been actively pursued [1,2,3]. The first sustained DDSs were synthesized in 1952 by coating the drugs with different layers of a polymer [4]. Nowadays, many studies are being conducted on triggered drug delivery, targeted drug delivery, and controlled drug delivery [5]. For developing long-term DDSs, biodegradable material can be used since removing them is not required after the total drugs are released [6]. Many DDSs have the ability to release drugs in response to internal stimuli such as enzymes, pH, and redox stimuli, while some DDSs are used with an external trigger such as hyperthermia and magnetic fields [7]. Some pH-trigger DDSs release the drug through conjugation to a nanocarrier having an acidic bond, including imine, orthoester, phosphoramidate, or hydrazone, which release the drug through hydrolyzing, while DDSs containing ionizable groups respond to different pH values through conformation or dissolution variation [8].

**Figure 1:**
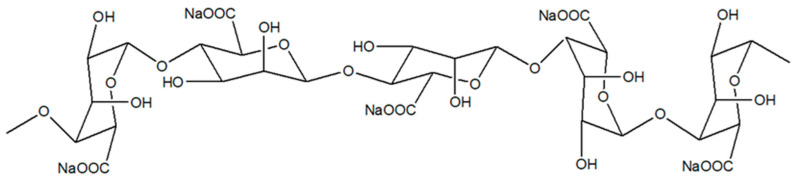
Chemical structure sodium alginate.

Hydrogel is a two- or multicomponent system containing a three-dimensional network of polymer chains, a cross-linked polymeric network, with the swelling and storing ability of a notable amount of water in its structure; however, it is not soluble in water [9,10,11,12]. It can be prepared using different methods: cross-linking, blending, in situ, and grafting. The cross-linking method has two types: chemical and physical cross-linking [13]. Hydrogels are appealing material systems that can be used as carriers for a broad range of therapeutics [14]. Polymers such as poly(ethylene glycol) [15] and poly(lactic-co-glycolic acid) [16] have been employed to form hydrogels utilized as drug carriers, offering many benefits including precise control of their physical and chemical properties, mechanical strength, and stability [17,18,19]. However, controlled drug release assurance remains challenging. Polymer science development is leading to biopolymer creation, which offers biocompatible and biodegradable hydrogels utilized for targeted and efficient drug delivery and enhance the physiological performance and tailor the functionality; natural polysaccharides can be used [20,21]. As an example, alginate, which is a polysaccharide, comes from the bacterial capsule of the *Azotobacter* species and the *Pseudomonas* species as well as the wall of brown algae [22]. Its constituents are 1,4-linked α-L-guluronic acid (G) and 1,4-linked β-D-mannuronic acid (M), the chemical structure of which alternates between both G and M components, repeating M and repeating G. Alginate, which gives strength in algal tissue and flexibility, is found in its natural conditions as alginic acid salts including sodium, calcium, and magnesium, as illustrated in Figure 1 [23,24]. The potential of using sodium alginate-based hydrogel in environmental protection and medicinal domains refers to the positive biocompatibility and biodegradability of this material, which has become a popular research area [25]. The low toxicity and degradability of sodium alginate-based hydrogels, as well as their uses in tissue engineering, drug release, and water treatment, show their significant value in sustainable development and green chemistry [26].

Particles in macro-to-micro range scale, beads, are suitable in targeted and sustained site-specific studies [27]. Many studies synthesized crosslinked alginate hydrogel beads to deliver different molecules. Camacho et al. used copper crosslinked alginate hydrogel for encapsulating and releasing folic acid [28]. Aldawsari et al. synthesized calcium crosslinked alginate hydrogel beads for loading apigenin and a release study afterward [29].

**Figure 2:**
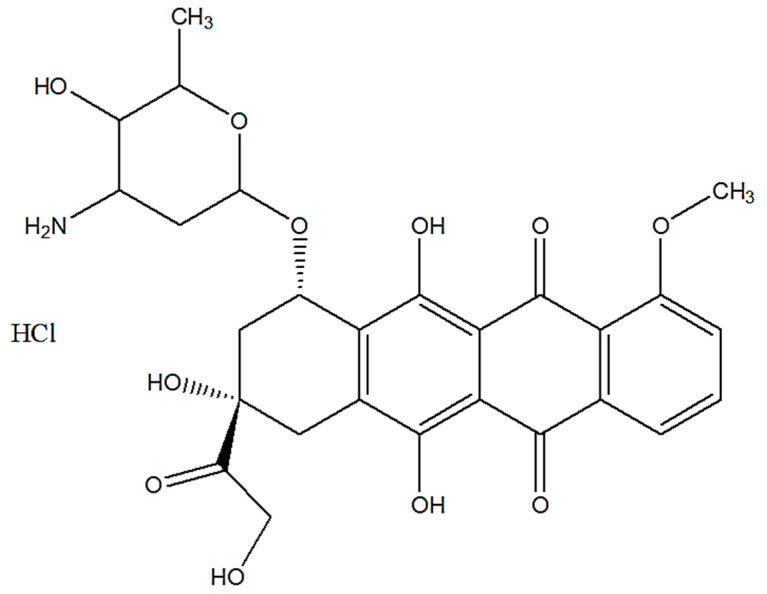
Chemical structure of doxorubicin hydrochloride.

Doxorubicin hydrochloride is an anthracycline ring antibiotic; its molecular formula is C_27_H_29_NO_11_·HCl, while its molecular structure is shown in Figure 2. It is used to treat cancers of the liver, lung, breast, ovary, bladder, thyroid, stomach, and leukemia; this is achieved by inhibiting the growth of cancer cells, which is called an anti-neoplastic agent [30,31]. However, cardiotoxicity, vomiting, alopecia, leucopenia, and stomatitis are the side effects of doxorubicin hydrochloride. Drug delivery vehicles were used to reduce these side effects [31] and to regulate the drug release rate into the body over time [32].

This research aims to incorporate doxorubicin hydrochloride into different crosslinked alginate beads and study the effect of the amount of crosslinked alginate, pH of solution, time, and concentration of doxorubicin hydrochloride on the drug loading, as well as examine how the pH of the solution affects the release of drugs. Doxorubicin hydrochloride was incorporated into different synthesized crosslinked alginate beads (Cupric alginate, Ferric alginate, Stannous alginate, Strontium alginate, and Nickel alginate). The type that has a high adsorption percentage was studied in detail. After that, research was performed on how the pH of the solution affects drug release.

## 2. Methodology

### 2.1. Chemicals and Materials

Sodium alginate (C_6_H_9_NaO_7_) was obtained from Sigma Aldrich (St. Louis, MI, USA), and ferric chloride hexahydrate (FeCl_3_·6H_2_O) was acquired from LobaChemie, Mumbai India. Stannous chloride dihydrate (SnCl_2_·2H_2_O) was obtained from Fluka (Gillingham, UK). Nickel chloride hexahydrate (NiCl_2_·6H_2_O) and cupric chloride 2-hydrate (CuCl_2_·2H_2_O) were obtained from BDH chemicals, Poole, England. Strontium chloride hexahydrate (SrCl_2_·6H_2_O) and doxorubicin hydrochloride (C_27_H_29_NO_11_·HCl) were acquired from Sigma Aldrich (St. Louis, MI, USA). During the entire experiment, distilled water was used.

### 2.2. Preparation of Crosslinked Sodium Alginate Hydrogel Beads

To form a sodium alginate solution (0.1 M, 100 mL), distilled water was mixed with sodium alginate powder (2 g, M/G = 1.56, molecular weight = around 12,000–40,000 Da); the heater was used (around 50–70 °C) to dissolve the alginate. A syringe was used to put dropwise the alginate solution into five different solutions: CuCl_2_·2H_2_O (0.6 M), FeCl_3_·6H_2_O (0.42 M), SnCl_2_·2H_2_O (0.4 M), NiCl_2_·6H_2_O (4.4 M), and SrCl_2_·6H_2_O (0.42 M); this was performed while stirring. After that, the beads were filtered from the solution by using an air pump and a filter paper with the addition of distilled water. Then, the beads were kept dry overnight.

### 2.3. Characterization

A scanning electron microscope (SEM of JEOL, JSM-IT-100, Tokyo, Japan) was applied to find out the surface morphology of ferric alginate beads. The same beads were examined using energy dispersive X-ray spectroscopy (EDS) for elemental analysis. The beads structure was confirmed using an X-ray diffraction spectrometer with Cu radiation, which was scanned throughout a 2θ range covering from 5° to 100°. A Fourier transform infrared (FT-IR) spectrometer of the Perkin Elmer spectrum 100 series (Beaconsfield, Bucks, UK) was used to obtain FT-IR spectra. The concentration of doxorubicin hydrochloride was quantified by a Thermo Scientific Evolution 350 UV-Vis spectrophotometer.

### 2.4. Adsorption Study

#### 2.4.1. Adsorption of Doxorubicin Hydrochloride by Different Types of Crosslinked Sodium Alginate Hydrogel Beads

A solution of doxorubicin hydrochloride (15.78 ppm) was prepared. By putting 10 mg of different types of cross-linked sodium alginate beads—ferric crosslinked sodium alginate (Fe(III)–Alg), cupric cross-linked sodium alginate (Cu(II)-Alg), stannous crosslinked sodium alginate (Sn-Alg), nickel cross-linked sodium alginate (Ni-Alg), and strontium cross-linked sodium alginate (Sr-Alg)—into 10 mL of five different conical flasks containing doxorubicin hydrochloride. By using the shaker, and after 24 h, the concentration of doxorubicin hydrochloride was checked. The adsorption percentage of doxorubicin hydrochloride was calculated from the plot of the UV-Vis spectrum utilizing the subsequent equation:

(1) $$\mathrm{Adsorption}\%\;=\frac{A_{○}-A_{t}}{A_{○}}\times 100$$

Whereas $A_{○}$ denotes the initial absorbance of doxorubicin hydrochloride at 488 nm ($\lambda_{max}$), and $A_{t}$ denotes the absorbance at time *t*.

The adsorption amount of doxorubicin hydrochloride on beads was calculated by the following equation:

(2) $$\mathrm{Adsorption}\;\mathrm{amount},\;q_{e}=\frac{\left\{C_{i}-C_{e}\right\}V}{m}$$

where m is the amount of beads (g), V is the volume of the doxorubicin hydrochloride solution, $C_{i}$ and $C_{e}$ are the initial and equilibrium concentrations of the doxorubicin hydrochloride in mg/L, respectively, and $q_{e}$ is the amount of doxorubicin hydrochloride adsorbed in mg/g at equilibrium.

#### 2.4.2. Effect of the Amount of Alginate Hydrogel Beads

10 mL of doxorubicin hydrochloride (15.78 ppm) was added to five different conical flasks. Then, various doses of Fe(III)-Alg beads (10, 25, 50, 75, and 100 mg) were added. After shaking for 24 h, the concentration of doxorubicin hydrochloride was checked.

#### 2.4.3. Effect of pH on the Adsorption Percentage

Three conical flasks were used to add 10 mL of doxorubicin hydrochloride (15.78 ppm) to each one. To change the solutions’ pH (pH 3.93, pH 7.46, and pH 8.99), HCl and KOH solutions were used. After that, 50 mg of Fe(III)-Alg beads were added to each solution. The concentration of doxorubicin hydrochloride was checked after 1, 2, 3, 4, 5, 6, 7, and 24 h; the shaker was used during the 24 h.

#### 2.4.4. Effect of Time on the Adsorption Percentage

A total of 50 mg of Fe(III)-Alg beads was added to 10 mL of doxorubicin hydrochloride (15.78 ppm). The solution was shaken for 24 h, and the concentration of the solution was checked after 1, 2, 3, 4, 5, 6, 7, and 24 h.

#### 2.4.5. Effect of Doxorubicin Hydrochloride Concentration on the Adsorption Percentage

First, 50 mg of Fe(III)-Alg beads was added to 10 mL of doxorubicin hydrochloride solutions of different concentrations (116.5, 81.59, 58.54, 30, and 19.56 ppm). After shaking for 24 h, the concentrations of the solutions were checked.

### 2.5. Drug Release Study

#### 2.5.1. Preparation of Doxorubicin Hydrochloride-Fe(III)-Alg

A total of four grams of Fe(III)-Alg was put into 400 mL of doxorubicin hydrochloride (43.46 ppm). After 24 h, the beads were removed and immersed in distilled water. Then, the beads were dried overnight.

#### 2.5.2. In Vitro Drug Release Study

We added 15 mg of doxorubicin hydrochloride-Fe(III)-Alg beads into different solutions, each one containing 10 mL of the solution of HCl (pH 1.52 and pH 4.55), and phosphate buffers (pH 6.85 and pH 7.39). After regular intervals of time, 2 mL of the solutions were removed and substituted with the equivalent amount of fresh media. The solutions were shaken for 25 h at 37 °C. By using the UV-Vis spectrophotometer, the drug absorbance was monitored at 488 nm.

#### 2.5.3. Drug Release Mathematical Modeling

Kinetic models were employed to evaluate the drug release kinetics and mechanism. The model that most closely matched the released data was chosen, which was thought to have the highest coefficient correlation (R^2^) value [33].

##### Zero Order Release Model

Zero order release model illustrated drug dissolution from pharmaceutical forms that do not disaggregate and release the drug slowly using the following formula:

(3) $$q_{t}={-k}_{0}t+q_{0}$$

Whereas $q_{t}$ denotes the concentration of doxorubicin hydrochloride at time *t*, $k_{0}$ denotes the zero order release constant, and $q_{0}$ denotes the concentration at time 0 [34].

##### First Order Release Model

Drug absorption and release can be described using this equation:

(4) $${\ln(q}_{t}/q_{0})={-k}_{1}t$$

Whereas $k_{1}$ denotes the first order release constant [34].

##### Hixson–Crowell Release Model

The following formula was derived by Hixson and Crowell, expressing the dissolution rate using the cubic root of particle weight, assuming the particle radius is not constant:

(5) $$\sqrt[{3}]{q_{t}}-\sqrt[{3}]{q_{0}})={-k}_{HC}t$$

Whereas $k_{HC}$ denotes the proportionality constant [34].

##### Higuchi Release Model

The drug dissolution from a planar system from a homogeneous matrix can be examined using the following formula:

(6) $$q_{t}=k_{H}t^{0.5}$$

Whereas $k_{H}$ denotes the Higuchi dissolving constant [34].

##### Bhaskar Release Model

This is a dissolution-diffusion kinetic release model, which is described by:

(7) $${\ln(q}_{t}/q_{0})=Bt^{0.65}$$

Whereas B denotes the appropriate kinetic constant [35].

## 3. Results and Discussion

**Scheme 1:**
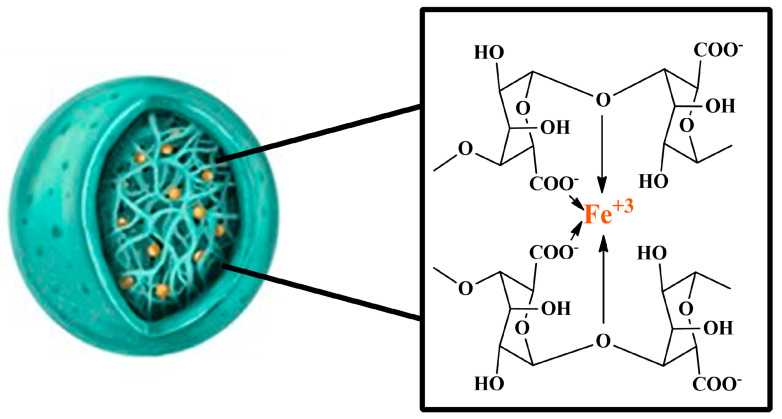
Schematic representation shows the crosslinking between Fe (III) and sodium alginate.

**Figure 3:**
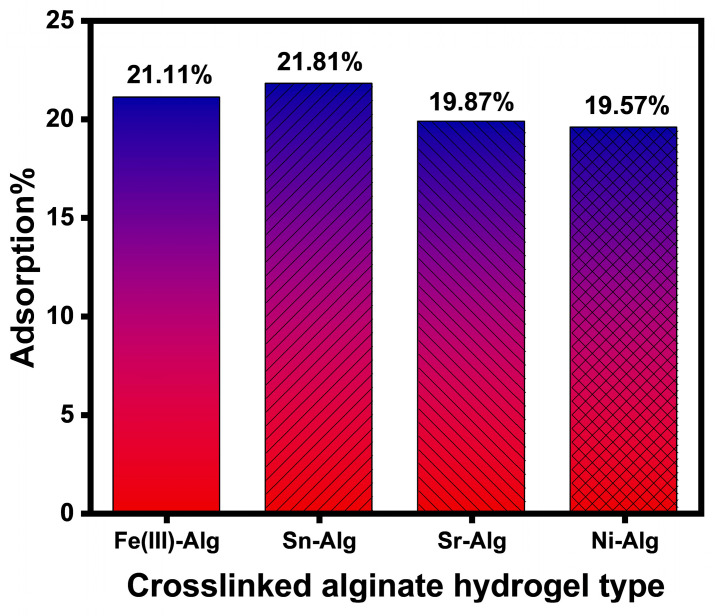
The adsorption percentage of doxorubicin hydrochloride by each type of crosslinked sodium alginate hydrogel.

Crosslinked sodium alginate hydrogel beads were synthesized successfully; Scheme 1 illustrates the chemical structure of Fe(III)-Alg. Fe(III)-Alg beads can be synthesized using the extrusion-dripping process, which involves a dropwise addition of a solution of sodium alginate into a solution containing iron(III) [36]. Assuming the crosslinking between Fe^+3^ and sodium alginate follows a model that proposes alginate coordinates Fe^+3^ to produce a spatially isolated Fe^+3^ center along with polysaccharide [37,38]. The affinity of alginate toward cations was not the same; some crosslinked with alginate using a diluted concentration solution, and some needed a more concentrated one, which agrees with the previous studies that showed a decrease in the manner of alginate affinity toward various divalent cations as follows: Pb > Cu > Cd > Ba > Sr > Ca > Ni, Zn, Co > Mn [39,40]. In Figure 3, a successful adsorption of doxorubicin hydrochloride on Fe(III)-Alg, Sn-Alg, Sr-Alg, and Ni-Alg is shown; it is obvious that Sn-Alg has a higher percentage of adsorption (21.81%), followed by Fe(III)-Alg with 21.11%. The focus on Fe(III)-Alg adsorption and release behavior is due to ligands giving stability and soft cationic features when Fe^+3^ forms complexes with polysaccharide ligands [41,42], where the binding between Fe^+3^ and alginate is stronger than that of other divalent and trivalent cations [43,44].

### 3.1. XRD Analysis

**Figure 4:**
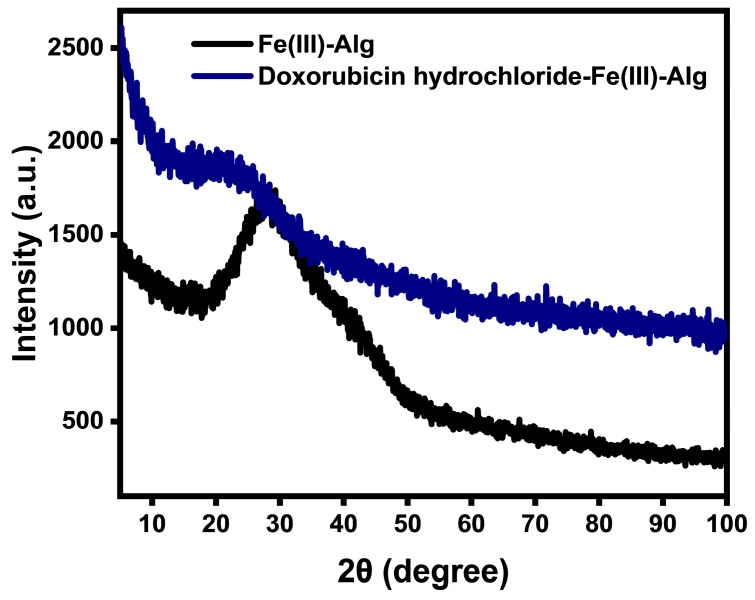
XRD pattern of Fe(III)–Alg (in black) and doxorubicin hydrochloride-Fe(III)–Alg (in blue).

The structural changes that occur during drug adsorption can be observed in Figure 4. The Fe(III)-Alg diffractogram exhibits a broad hump around 20–35°, which is characteristic of an amorphous polymeric structure resulting from Fe(III) crosslinking within the alginate matrix. The Fe(III)–Alg matrix is primarily amorphous, as confirmed by the absence of sharp diffraction peaks. After adsorption of doxorubicin hydrochloride, the diffractogram shows a slight shift and a decrease in the intensity of the broad peak, indicating molecular interactions between the drug and the Fe(III)–Alg network through hydrogen bonding, electrostatic attraction, or coordination with Fe^3+^ centers. Similar observations have been reported by Mandal et al. [45]. The lack of distinct crystalline peaks attributed to doxorubicin hydrochloride, as observed in earlier investigations [46], supports effective adsorption and encapsulation of the drug inside the Fe(III)-Alg matrix without the aggregation of discrete crystalline doxorubicin hydrochloride.

### 3.2. FTIR Analysis

**Figure 5:**
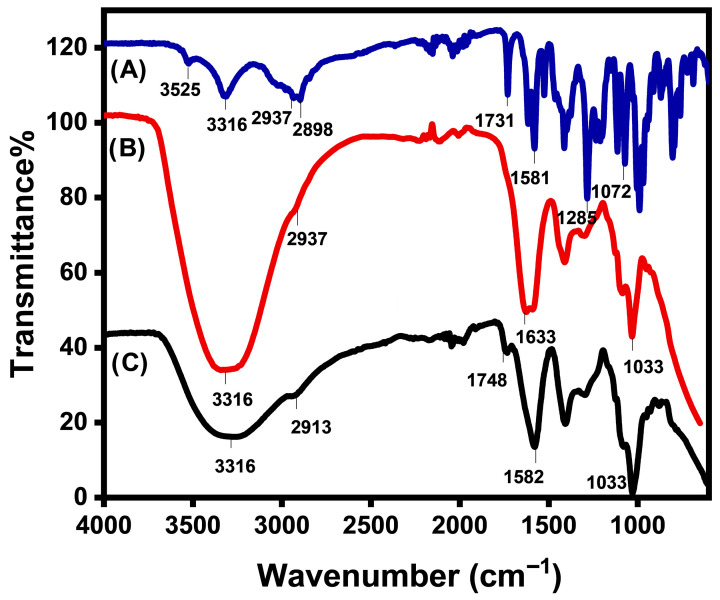
FTIR spectra of (**A**) doxorubicin hydrochloride, (**B**) Fe(III)–Alg beads, and (**C**) doxorubicin hydrochloride-Fe(III)–Alg beads.

FTIR spectra present the adsorption of doxorubicin hydrochloride onto Fe(III)–Alg. The spectra of doxorubicin hydrochloride are displayed in Figure 5A, showing the characteristic bands of NH_2_, OH, CH (alkene), CH (alkane), C=O, C=C, C-N, and C-O at 3525, 3316, 2937, 2898, 1731, 1581, 1285, and 1072 cm^−1^, respectively. The spectra of Fe(III)–Alg, Figure 5B, have bands at 3316, 2937, 1633, and 1033 cm^−1^ for OH, CH (alkane), C=O, and C-O functional groups, respectively. The spectra in Figure 5C confirms the adsorption of doxorubicin hydrochloride by appearing a band of C=O at 1748 cm^−1^.

### 3.3. SEM Analysis

**Figure 6:**
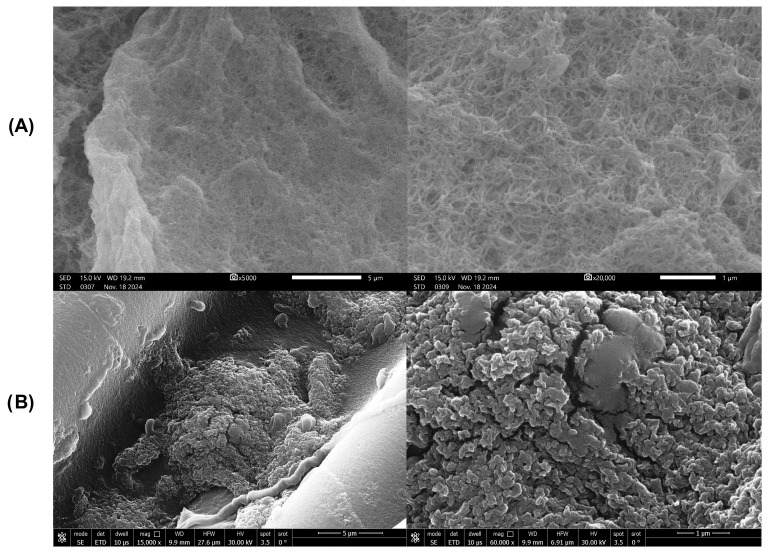
(**A**) SEM of Fe(III)–Alg beads. (**B**) SEM of doxorubicin hydrochloride–Fe(III)–Alg beads.

The SEM technique gives information about surface morphology. The SEM photograph in Figure 6A shows the high porosity of the surface of Fe(III)–Alg beads, which indicates its adsorption efficiency; this is due to the extended cross-linking of alginate with Fe^3+^ ions [47]. A study agrees with this finding, which shows the surface of Fe(III)–Alg hydrogel has a high distribution of macropores [48]. After doxorubicin hydrochloride adsorption (Figure 6B), the pores disappeared.

### 3.4. EDS Analysis

**Figure 7:**
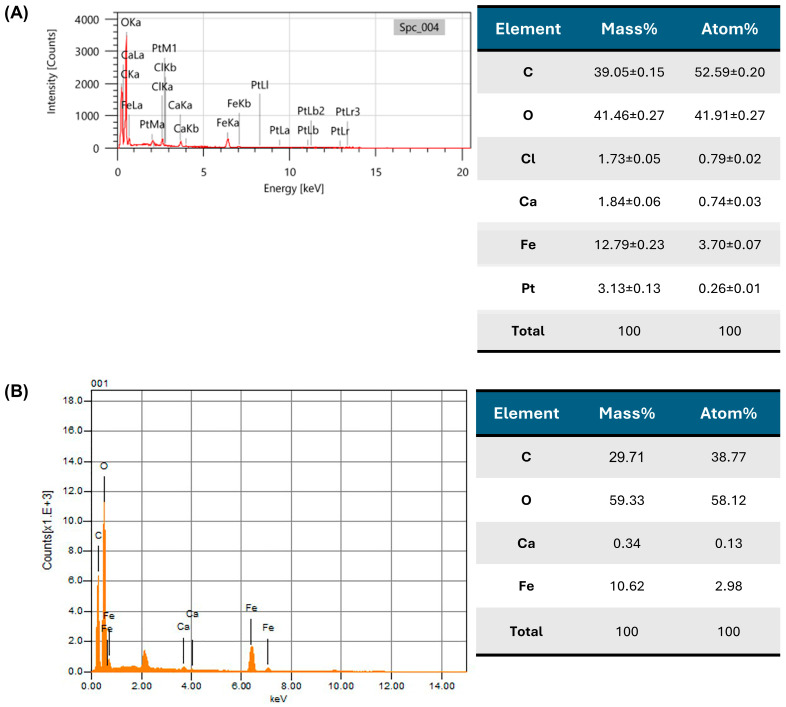
EDS of (**A**) Fe(III)–Alg and (**B**) doxorubicin hydrochloride-Fe(III)–Alg beads.

The EDS spectrum confirms that C, O, and Fe are the main elements of Fe(III)–Alg with 39.05, 41.46, and 12.79 of the mass %, respectively, as shown in Figure 7A. The same is true for doxorubicin hydrochloride–Fe(III)–Alg, Figure 7B, since mass percentages of C, O, and Fe are 29.71%, 59.33%, and 10.62%, respectively.

### 3.5. Adsorption Behavior

**Figure 8:**
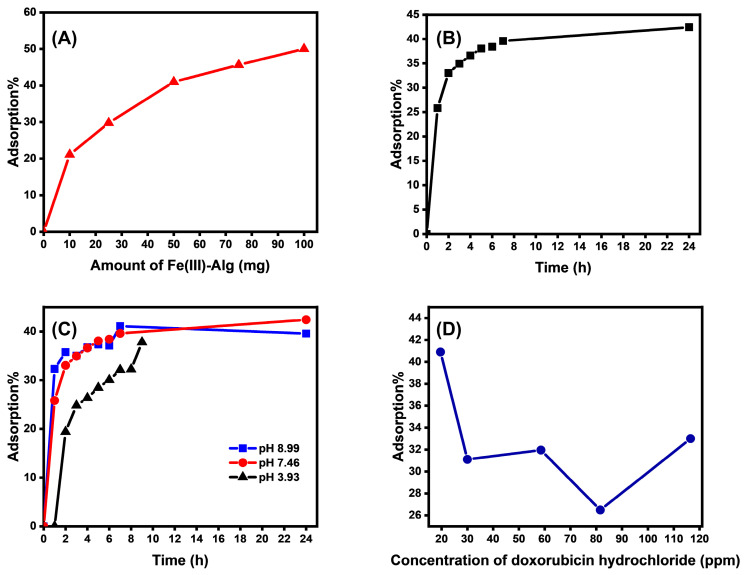
(**A**) Fe(III)–Alg dose effect, (**B**) time effect, (**C**) solution’s pH effect, and (**D**) doxorubicin hydrochloride concentration effect on the drug’s adsorption.

Fe(III)–Alg beads were tested and assessed for different parameters for maximum adsorption of doxorubicin hydrochloride. The adsorption % presented in Figure 8A increased with increasing the amount of Fe(III)–Alg beads and time, to assess the effects of Fe(III)–Alg beads quantity on adsorption of doxorubicin hydrochloride. The evaluation of the suitable amount of Fe(III)–Alg is the crucial factor in the adsorption process since it significantly regulates the mass transfer of doxorubicin hydrochloride from the solution to the Fe(III)–Alg. The influence of Fe(III)–Alg amount was checked by using 10–100 mg while keeping other parameters constant. It was deduced that with an increase in Fe(III)–Alg beads amount, the adsorption was increased; 10 mg of Fe(III)–Alg beads gave the least adsorption of doxorubicin hydrochloride, while 100 mg achieved the highest adsorption of doxorubicin hydrochloride. Likewise, Wu et al. [49] studied doxorubicin hydrochloride adsorption properties on graphene oxide and found the adsorption percentage increased by increasing the dose of graphene oxide, which resembles the effect of adsorbent dose in this study.

The impact of contact time was examined to assess the adsorption of doxorubicin hydrochloride onto the Fe(III)-Alg beads. Figure 8B illustrates that initially there is rapid adsorption of doxorubicin hydrochloride which then gradually increases with time. The adsorption of doxorubicin hydrochloride does not significantly change with more time. According to these findings, doxorubicin hydrochloride was most effectively adsorbed by Fe(III)–Alg beads after 24 h. First, a large number of many accessible adsorption sites on Fe(III)–Alg beads are responsible for the high adsorption of doxorubicin hydrochloride. The adsorption capacity of Fe(III)–Alg beads was 3.897 mg/g for doxorubicin hydrochloride. These findings show that there were many available unoccupied sites, which led to a high adsorption. However, the repulsive force of the already adsorbed doxorubicin hydrochloride made it more difficult to access the remaining sites. These findings verify that the majority of the vacant sites on the surface of Fe(III)–Alg beads can be occupied in the first 60 min. pH of doxorubicin hydrochloride solution is the primary factor that affects the adsorption on Fe(III)–Alg beads. The pH of the doxorubicin hydrochloride solution protonates or deprotonates the drugs as well as modifies the surface of Fe(III)-Alg beads and the adsorption affinity of the drug. Figure 8C shows slight differences in the percentage adsorption between different pH solutions of doxorubicin hydrochloride since it was the best when the pH of doxorubicin hydrochloride was 7.46. However, at pH 3.39 and pH 8.99, the adsorption was slow and ended with nearly the same percentage as it was at pH 7.46. Doxorubicin is in the anionic form at high pH and in the cationic form at low pH [50,51]. The previous study, Wu et al., gave the highest adsorption percentage at pH 8.5 [49]. The results illustrate that at low pH, adsorption of doxorubicin hydrochloride on Fe(III)–Alg beads was minimal. However, the adsorption of doxorubicin hydrochloride on Fe(III)–Alg beads rose continuously as the pH of the solution increased, reaching its maximum at pH 7.46.

The evaluation of appropriate initial concentrations of doxorubicin hydrochloride solution is a crucial parameter in adsorption research since it significantly regulates the mass transfer of drug from the drug solution to the adsorbent, so it is important to evaluate the effects of doxorubicin hydrochloride concentration on the adsorption process. Using varying concentrations of doxorubicin hydrochloride while maintaining all other parameters constant, the impact of the initial drug concentration was examined. It was deduced that with an increase in doxorubicin hydrochloride concentration in the solution, adsorption decreased. The drug was adsorbed in high amounts when its concentration was 19.56 ppm (Figure 8D). Thus, the optimum concentration of doxorubicin hydrochloride was 19.56 ppm. Further increase in doxorubicin hydrochloride concentration was decreased and gave less adsorption.

### 3.6. Release Behavior

**Figure 9:**
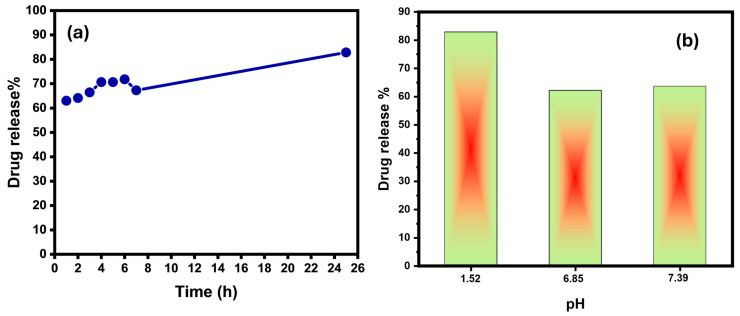
Doxorubicin hydrochloride released (**a**) at pH 1.52 as a function of time and (**b**) at different pH.

The release profiles of the doxorubicin hydrochloride from Fe(III)–Alg depends on the molecular interaction between doxorubicin hydrochloride and Fe(III) which can greatly influence the release patterns. A sustained drug release was tested at pH 1.52 for 25 h period, as illustrated in Figure 9a. Initially, burst release was observed after 1 h with 63.006%, which increased gradually to 82.822% after 25 h. The release fluctuated at pH 6.85 and at pH 7.39 (Figure 9b), while at pH 4.55, there was no release. The release did not exceed 82.822% and was not lower than 62.108% throughout the different pH. This dissolution test suggested a sustainable in vitro release of the drug in the gastric fluid, and an inconsistent release in the intestinal fluid [49,52]. A summary of this study release percentage and the previous studies for the delivery of doxorubicin hydrochloride after one day is provided in Table 1.

### 3.7. Kinetic Study

**Figure 10:**
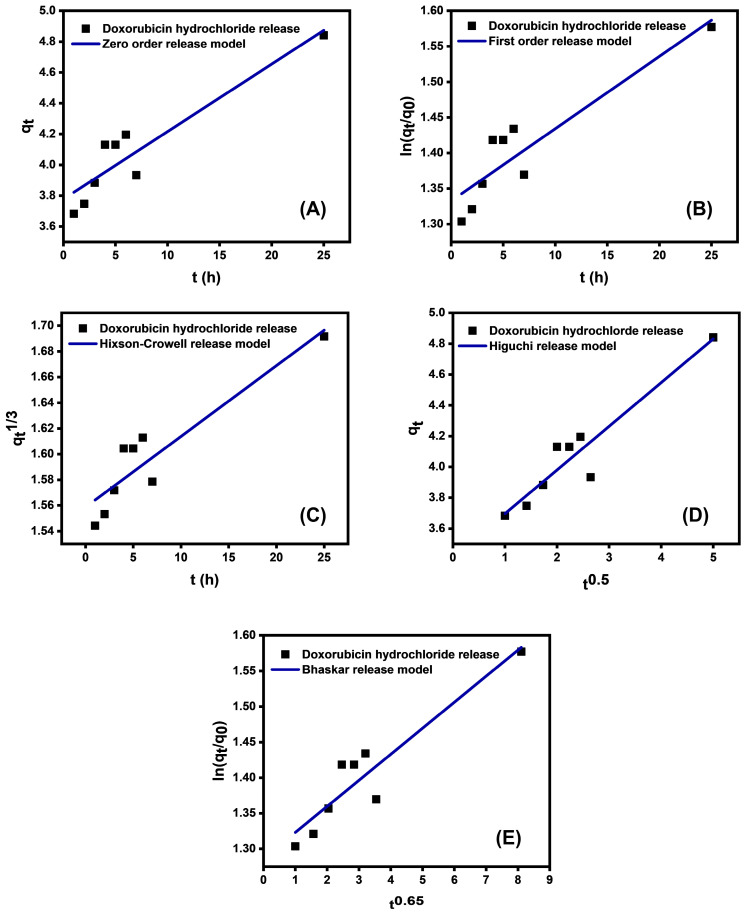
Fitting of doxorubicin-Fe(III)-Alg at pH 1.52 using various models of kinetic: (**A**) Zero order, (**B**) First order, (**C**) Hixson–Crowell, (**D**) Higuchi, and (**E**) Bhaskar release models.

To examine the release kinetics at pH 1.52, this study used kinetic models as follows: zero order, first order, Hixson–Crowell, Higuchi, and Bhaskar (Figure 10). The linear correlation coefficient (R^2^) is provided in Table 2; the coefficient R^2^ had the greatest value of 0.89731 using the Higuchi kinetic release model. Al-Shanqiti et al. synthesized promethazine hydrochloride/chitosan (PMT/CH) hydrogel, which released PMT at pH 7.4, giving the highest R^2^ value by the Higuchi release model [56]. For Amitriptyline/chitosan (AMT/CH) hydrogel, AMT was released at pH 6.8, which was best fitting the Higuchi model, which has an R^2^ = 0.99567 [57]. The same for nanohybrid of cefuroxime (CFO) since releasing from the layered double hydroxide-CFO (LDH-CFO) system showed that the Higuchi model was the most appropriate [58]. This model explicates the drug release kinetics of various pharmaceutical forms (such as porous matrix) using pseudo-steady state assumptions in light of Fick’s law, which shows time-dependent process’ square root [59]. Hence, this drug delivery system follows Fick’s diffusion mechanism, which means that it is a diffusion-controlled drug delivery system [60]. In diffusion-controlled DDSs, the drug release is controlled through diffusion from hydrogel pores or mesh filled with water [61]. However, several circumstances need to exist: the diffusion must be constant and unidirectional, the concentration of the initial drug needs to be higher than the drug’s saturation condition in the designated matrix, and the matrix’s dissolution and any border’s effect should be trivial [56,62].

## 4. Conclusions

To sum up, this research synthesized different types of crosslinked sodium alginate hydrogel beads (Fe(III)–Alg, Sn–Alg, Sr–Alg, Ni–Alg, and Cu(II)–Alg) and studied their adsorption toward doxorubicin hydrochloride. Fe(III)–Alg and Sn–Alg showed high adsorption of doxorubicin hydrochloride. Therefore, Fe(III)–Alg beads were thoroughly characterized. SEM photograph of Fe(III)–Alg confirmed its adsorption efficiency, and EDS showed the main components. The adsorption % was the best when the amount of Fe(III)–Alg was 100 mg, after 24 h, at pH 7.46, and the concentration of doxorubicin hydrochloride was 19.56 ppm. The drug was released from doxorubicin hydrochloride–Fe(III)–Alg beads for 25 h at pH 1.52, which simulates the gastric fluid, thereby making it suitable for oral use. The percentage of the drug release after one day was 82.822%, assuming release following the Higuchi model. Future in-vivo research needs to verify the biocompatibility of the doxorubicin–Fe(III)–Alg system.

## Figures and Tables

**Table 1:** Summary of the release of doxorubicin hydrochloride from different carriers after one day.

Drug Carrier	pH	Release%	Reference
Doxorubicin hydrochloride–Fe(III)-Alg	1.52	82.822%	Current study
	6.85	62.108%	
ALD-NS+DOX (Aldehyde-functionalized nanosponge nanoparticle-doxorubicin hydrochloride)	5.2 (NaOAc buffer)	64.6%	[53]
	5.2 (PBS buffer)	62.4%	
CNHN I (Chitosan nanohydrogel networks I)	5	56%	[54]
	7.4	5%	
HA/Zein-DOX NPs (Hydroxyapatite/Zein-doxorubicin hydrochloride nanoparticles)	7.4	28.1%	[55]
Zein-DOX NPs	7.4	61.8%	[55]

**Table 2:** Correlation coefficients of the kinetic models used for the release of doxorubicin hydrochloride at pH 1.52.

Kinetic Release Model	R^2^
Zero order	0.85869
First order	0.83067
Hixson-Crowell	0.84037
Higuchi	0.89731
Bhaskar	0.87306

## Data Availability

Data are contained within the article.
